# Association of serotonin receptor gene polymorphisms with anorexia nervosa: a systematic review and meta-analysis

**DOI:** 10.1007/s40519-024-01659-3

**Published:** 2024-04-26

**Authors:** Arturo Bevilacqua, Francesca Santini, Daniela La Porta, Silvia Cimino

**Affiliations:** 1 Department of Dynamic, Clinical Psychology and Health Studies, Sapienza University of Rome, Via Dei Marsi 78, 00185 Rome, Italy; 2 Systems Biology Group Lab and The Experts Group on Inositols in Basic and Clinical Research (EGOI), Research Center in Neurobiology Daniel Bovet (CRiN), Rome, Italy; 3 Department of Psychology of Development and Socialization Processes, Sapienza University of Rome, Via Dei Marsi 78, 00185 Rome, Italy; 4 Department of Psychology, Sapienza University of Rome, Via Dei Marsi 78, 00185 Rome, Italy

**Keywords:** Anorexia nervosa, Candidate gene studies, Serotonin, *5-HT2A*, *5-HTR2A*, *5-HT2C*, *5-HTR2C*

## Abstract

**Purpose:**

Several studies have investigated the association between anorexia nervosa and polymorphisms of genes regulating serotonin neurotransmission, with a focus on the rs6311 polymorphism of *5-HTR2A*. However, inconsistent results of these studies and conflicting conclusions of existing meta-analyses complicate the understanding of a possible association. We have updated these results and evaluated the involvement of other serotonin receptor gene polymorphisms in anorexia nervosa.

**Methods:**

Adhering to PRISMA guidelines, we have searched studies on anorexia nervosa and serotonin-regulating genes published from 1997 to 2022, selected those concerning receptor genes and meta-analyzed the results from twenty candidate gene studies on the *5-HTR2A* rs6311 polymorphism and the *5-HTR2C* rs6318 polymorphism.

**Results:**

Present analyses reveal an association for the *5-HTR2A* rs6311 polymorphism, with G and A alleles, across eighteen studies (2049 patients, 2877 controls; A vs. G allele, Odds Ratio = 1.24; 95% Confidence Interval = 1.06–1.47; p = 0.009). However, after geographic subgrouping, an association emerged only in a Southern European area, involving five studies (722 patients, 773 controls; A vs. G allele, Odds Ratio = 1.82; 95% Confidence Interval = 1.41–2.37; p < 0.00001). No association was observed for the *5-HTR2C* rs6318 polymorphism across three studies*.*

**Conclusions:**

To date, the involvement in the pathophysiology of anorexia nervosa of the *5-HTR2A* rs6311 polymorphism appears limited to a specific genetic and/or environmental context, while that of the *5-HTR2C* rs6318 polymorphism seems excluded. Genome-wide association studies and epigenetic studies will likely offer deeper insights of genetic and environmental factors possibly contributing to the disorder.

**Level of evidence:**

III Evidence obtained from well-designed cohort or case–control analytic studies.

*Clinical trial registration* PROSPERO registration number: CRD42021246122.

## Introduction

Eating disorders (EDs), including anorexia nervosa (AN), bulimia nervosa (BN) and binge eating disorder (BED), are potential life-threatening multidimensional syndromes classified in the *Diagnostic and statistical manual of mental disorders* (DSM-5-TR, [1]). Although their pathogenesis is still unclear, a widely accepted bio-psycho-social model implicates psychological, familial, socio-cultural, and genetic factors. A significant genetic influence is suggested by family, twin, and molecular genetics studies [2–8]. An individual susceptibility to Eds is also acknowledged in the psychodynamic framework, coupled to the importance of family functioning in their onset and maintenance, especially in adolescents [9–12].

AN, the most thoroughly investigated ED, predominantly affects females with a lifetime prevalence of 0.2–3% [13–15]. It is characterized by a morbid fear of gaining weight that leads to food avoidance [13] and is associated with factors such as cognitive impairments [16] including poor information processing, rigid thinking patters and attentional bias on memory encoding and recall [17].

Over the past three decades, the genetics of AN has been investigated by various approaches including candidate gene studies, linkage analyses, transmission disequilibrium tests (TDT), genome-wide association studies (GWAS), and epigenetic studies, each one bearing distinct advantages and limitations [5, 18, 19]. Candidate gene studies began in the late 1990s exploring the association between specific genes and AN. Among the neurochemical systems investigated, the serotonergic system has received considerable attention due to its role in regulating mood, food intake, and body weight [20], and to alterations in its functioning directly linked to the etiology of AN [21].

In particular, several studies have focused on the rs6311 single nucleotide polymorphism (SNP) of the *5-HT2A* receptor gene *(5-HTR2A),* with G and A alleles, and other receptor gene polymorphisms, including the rs6318 SNP of the *5-HT2C* receptor gene *(5-HTR2C),* with Cys and Ser alleles*.*

However, many of these studies have shown limitations, leading to frequent inconsistent results and impeding a thorough understanding of the roles of the analyzed polymorphisms [22, 23]. Limiting issues include small sample sizes, low-level replication, geographic variations in genotype/allele frequencies, and population stratifications. Additionally, the intrinsic complexity of AN, lacking a clear biological definition, suggests that its neurobiological etiology is influenced by the activity of multiple genes and significant gene x environment interactions. These factors cast doubt on the reliability of results of these studies.

Previous meta-analyses have focused on studies concerning the *5-HTR2A* rs6311 SNP. By analysis of six early studies [24–29], including 556 patients and 1077 controls, Ziegler et al. [29] found no association with AN and high genetic heterogeneity of the samples. In contrast, Gorwood et al. [30] reported a small yet significant association (A vs. G allele, Odds Ratio [OR] = 1.20; 95% Confidence interval [CI] 1.07–1.35; χ^2^ = 8.14; p = 0.0043), after incorporating three subsequent studies [31–33] with 872 patients and 1656 controls. However, this was coupled to a high heterogeneity (χ^2^ = 41.7; p = 1.51 × 10^–6^) that was attributed to a lack of statistical power, variations in allele frequencies among control samples, and methodological differences among the studies. Additionally, a slightly stronger association emerged in the studies involving Caucasian participants (OR = 1.27; 95% CI 1.12–1.44; p = 0.0003), but the small number of reports prevented a deeper examination of geographical differences.

Martásková et al. [34] reported a similar association (A vs. G allele, OR = 1.21; 95% CI 1.09–1.35; p < 0.003), after incorporating two additional studies [34, 35], totaling 1057 patients and 1599 controls. The authors did not provide heterogeneity data but investigated the geographical distribution of European cohorts. Positive association trends were identified in British and Italian cohorts, a Polish cohort and a U.S.A. cohort, but not other European and Asian cohorts, underlining the importance of considering population stratification in further assessments.

In a recent meta-analysis of previous reports and four additional studies [36–39], with 2028 patients and 2725 controls, Yan et al. [40] found a small global association (A vs. G allele, OR = 1.24; 95% CI 1.04–1.48; p = 0.014) and a more significant one in a subgroup of twelve studies on Western samples (A vs. G allele, OR = 1.31; 95% CI 1.11–1.64; p = 0.003), with high heterogeneity in both cases. In contrast, no association and low heterogeneity were observed in a subgroup of three studies on East Asian samples. The authors did not conduct further subgroup analyses.

Due to weaknesses of the existing meta-analyses, including the incorporation of studies that contained virtual controls or samples calculated from TDTs, and the lack of statistical corrections necessary under multiple genetic models, these results cast doubt on the presence of a *bona fide* association between the *5-HTR2A* rs6311 polymorphism and AN.

We have therefore reassessed the findings of serotonin candidate gene studies on AN by an updated and comprehensive systematic review and meta-analysis. Upon evaluation of all reports by the criteria of quality assessment, geographic location, participants’ age and gender, diagnosis, AN subtypes, we performed an analysis whose significant discriminating factor is a detailed geographic evaluation of effect sizes, accompanied by statistical correction for multiple testing.

## Methods

### Article search and selection

Searches for publications were conducted in PubMed (PubMed, https://pubmed.ncbi.nlm.nih.gov/, last accessed August 16th, 2023), Scopus (Scopus, https://www.scopus.com/search/form.uri#basic, last accessed June 21st, 2023), PsycINFO (PsycINFO, https://web.p.ebscohost.com/ehost/search/ advanced?vid = 2&sid = f36c3704-5378-4d93-8629-ac1da74db433%40redis, last accessed June 12th, 2023) and Web of Science (Web of Science, https://www-webofscience-com.ezproxy.uniroma1.it/wos/woscc/basic-search, last accessed June 20th, 2023) databases following PRISMA guidelines [41], from 1997, when the first candidate gene studies emerged, to December 2022. We initially used the terms “genetic association” AND “anorexia nervosa”, obtaining the following numbers of publications: PubMed: 578, Scopus: 552, PsycINFO: 42, Web of Science: 450. We subsequently used the terms “gene association” AND “anorexia nervosa”, obtaining the following numbers of publications: PubMed: 353, Scopus: 446, PsycINFO: 52, Web of Science: 478.

More specific searches, conducted using the terms “serotonin” AND “genetic association” AND “anorexia nervosa”, and the terms “serotonin” AND “gene association” AND “anorexia nervosa”, yielded the following respective numbers of publications: PubMed: 42, Scopus: 124, PsycINFO: 9, Web of Science: 86; PubMed: 60, Scopus: 109, PsycINFO: 21, Web of Science: 125.

After merging the search results and excluding duplicates, 222 papers were reviewed. Ninety non-genetic papers, papers not concerning serotonin or AN, papers lacking useful genetic information, and 8 papers involving animal models were excluded. The remaining 124 publications were selected by the following criteria:

- Study type: we included population-based, case–control genetic association studies. We excluded family- and patient-based studies, TDT studies, mutation analyses, association and/or haplotype analyses, GWAS;
- Diagnosis: we included studies in which AN was diagnosed according to the DSM-III-R [42], DSM-IV [43], DSM-IV-TR [44], DSM-5 [45] or ICD-10 [46] and control participants had no diagnosis of any pathology. We excluded studies in which participants were classified by administered or self-administered questionnaires;
- Polymorphism type: we included studies that investigated bi-allelic polymorphisms;
- Numerosity: we analyzed genetic polymorphisms investigated in at least three independent studies [47].

**Fig. 1:**
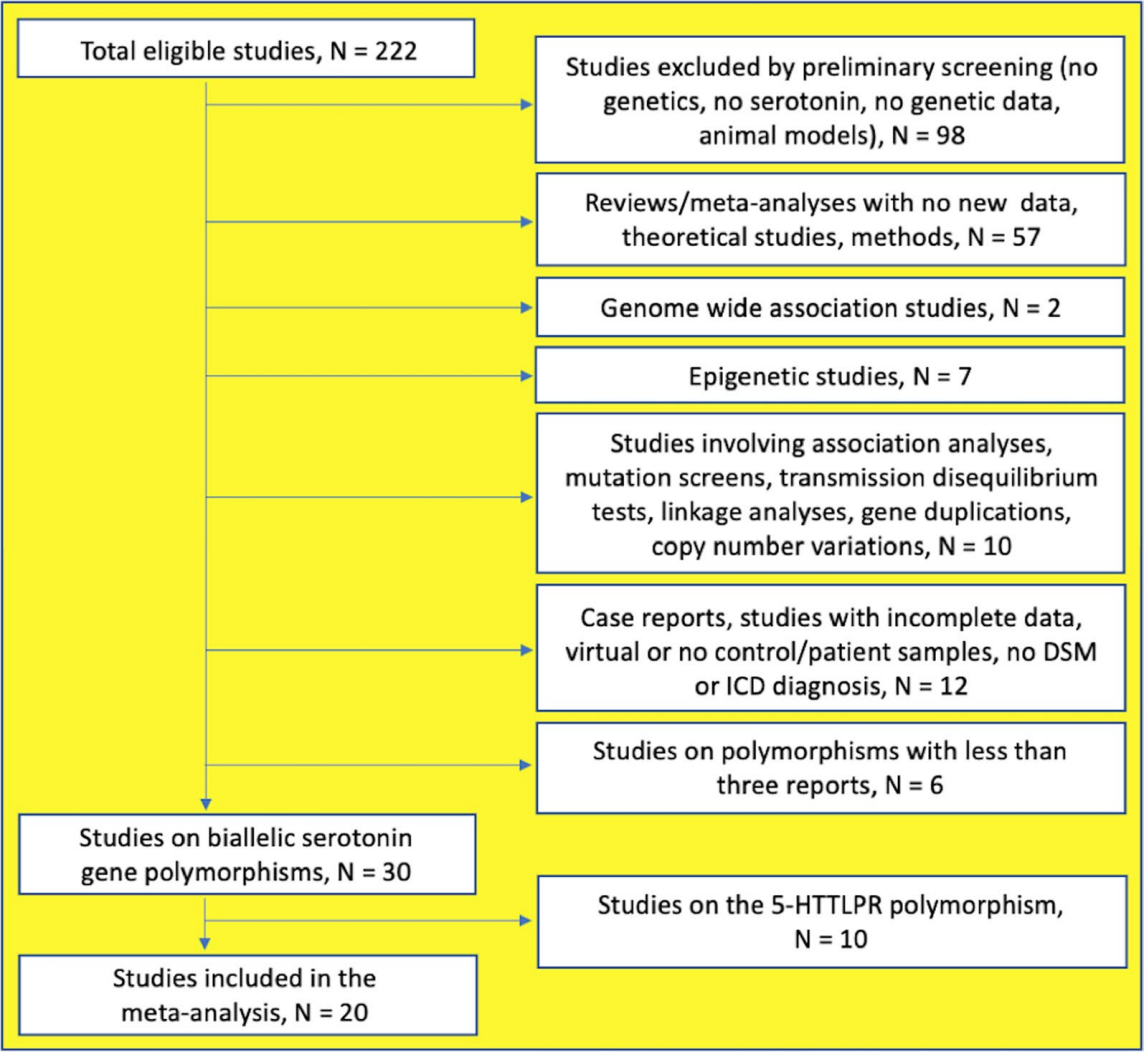
PRISMA flow of study selection

The search and selection of papers were conducted independently by F. Santini and D. La Porta, reviewed and approved by all authors. Based on the listed criteria, we excluded 52 reviews or methods/perspective papers, 5 out of 8 meta-analyses lacking empirical data, 2 GWAS papers, 7 epigenetic papers; 8 gene mapping, linkage, and mutation analyses, 2 TDTs, 8 papers with no control/patient samples or with virtual control samples, calculated from non-transmitted alleles in the family trios analyzed in TDTs, 2 papers without DSM/ICD diagnoses, 2 case-report papers, 6 papers not meeting the numerosity criterion. The remaining 30 papers on biallelic serotonin gene polymorphisms, including 27 research papers and 3 papers with research and meta-analytical results were further selected. (Fig. 1). After exclusion of 10 papers on the 5-HTTLPR polymorphism of the presynaptic serotonin transporter gene, 20 papers on *5-HTR2A* and *5-HTR2C* SNPs were meta-analyzed.

All papers were in English except for one Polish paper, which however had an English abstract and clear genotype frequencies.

### Meta-analysis

For all studies, selection, comparability and exposure qualities were assessed by the Newcastle–Ottawa quality rating scale (NOS) [48]; publication bias was evaluated by the Egger’s test [49]; Hardy–Weinberg (HW) equilibrium was examined by χ^2^-test.

Data collection from each study was performed independently by F. Santini and D. La Porta, reviewed and approved by all authors. The association of each genetic polymorphism with AN was evaluated by separate analyses for categorical variables. Genotype frequencies of patients and control participants from each study were examined using recessive (AA vs. Aa + aa), dominant (AA + Aa vs. aa), and allele (A vs. a) models, which effectively identify SNP risks in case–control genetic studies, based on mathematical evidence [50]. Additive, co-dominant and over-dominant models were therefore not considered. To control for family-wise error rate, Bonferroni correction was applied to account for multiple hypothesis tests related to our three genetic models (p = 0.05/3) [51]. Thus, *P* values ≤ 0.0166 were considered statistically significant.

Heterogeneity among the studies was assessed using a χ^2^-based *Q*-test [52] and quantified by *I*^2^ statistics [53]. Associations were analyzed by calculating the pooled OR and 95% CI, using the Mantel–Haenszel fixed effects (FE) model [54] for studies with low heterogeneity (p > 0.1 or *I*^2^ < 50.0%), or the random effects (RE) model for studies with high heterogeneity (p ≤ 0.1 or *I*^2^ ≥ 50.0%) [55]. The significance of the pooled OR was determined using a Z-test. The magnitude of the ORs was evaluated by Cohen’s criteria [56] as follows: OR < 1.44: very small effect; 1.44 ≤ OR < 2.48: small effect; 2.48 ≤ OR < 4.27: medium effect; OR ≥ 4.27: large effect.

If *I*^2^ exceeded 50.0%, the stability of the relationship was assessed by conducting analyses after removing one study at a time (leave-one-out test). Geographical distribution of the studies was considered to account for population stratifications. Subgrouping criteria based on gender, age, DSM version, NOS, were not applied for the following reasons: most studies included only or predominantly female participants; age distribution, when disclosed, did not significantly differ across the studies, the DSM-IV version was predominantly used, and NOS values were largely consistent. Analyses were performed using Review Manager 5.3 (The Nordic Cochrane Centre, Copenhagen, DM) and MedCalc 20.211 (MedCalc Software Ltd, Ostend, Belgium).

## Results

Serotonergic genes investigated in AN in association analyses, TDTs and case–control studies include *tryptophan hydroxylase 1 and 2 (TPH1* and *TPH2),* regulating serotonin biosynthesis, the presynaptic transporter *5-HTT,* and *5-HTR1D, 5-HTR2A, 5-HTR2C, 5-HTR3A, 5-HTR3D, 5-HTR7* receptors. Among serotonin receptor gene polymorphisms (Table 1), *5-HTR2A* rs6311 and *5-HTR2C* rs6318 met our inclusion criteria. Numbers of case–control associations reports are listed in Table 2, their genotyping methods, diagnostic criteria, and NOS values are provided in Table 3. Significance values of publication biases assessed by the Egger’s test are presented in figure legends.

**Table 1:** Serotonin receptor gene polymorphisms investigated in the period 1997–2020 and inclusion/exclusion criteria of the present meta-analysis

Author	Year	Gene	Polymorphism	Included/not included	Criterion for exclusion
Hinney et al.	1997	5-HTR2A	rs63311	Yes	
Collier et al.	1997	5-HTR2A	rs63311	Yes	
Campbell et al.	1998	5-HTR2A	rs63311	Yes	
Enoch et al.	1998	5-HTR2A	rs63311	Yes	
Sorbi et al.	1998	5-HTR2A	rs63311	Yes	
Nacmias et al.	1999	5-HTR2A	rs63311	Yes	
Ziegler et al.	1999	5-HTR2A	rs63311	Yes	
Hinney et al. https://doi.org/10.1038/sj.ijo.0800926	1999	5-HT1RDbeta	Phe-124-Cys	No	Numerosity
Hinney et al. https://doi.org/10.1038/sj.ijo.0800926	1999	5-HTR7	Pro-279-Leu	No	Numerosity
Ando et al.	2001	5-HTR2A	rs63311	Yes	
Karwautz et al.	2001	5-HTR2A	rs63311	Yes	
Karwautz et al.	2001	5-HTR2C	rs6318	Yes	
Nishiguchi et al.	2001	5-HTR2A	rs63311	Yes	
Ricca et al.	2002	5-HTR2A	rs63311	Yes	
Kipman et al.	2002	5-HTR2A	rs63311	Yes	
Westberg et al.	2002	5-HTR2C	rs6318	Yes	
Gorwood et al. https://doi.org/10.1038/sj/mp/4000938	2002	5-HTR2A	rs63311	No	TDT
Rybakowski et al.	2003	5-HTR2A	rs63311	Yes	
Hu et al.	2003	5-HTR2C	rs6318	Yes	
Bergen et al. https://doi.org/10.1038/sj.mp.4001318	2003	5-HTR1D	rs652783 rs604030 rs674386 rs856510	No	Linkage analysis
Ricca et al.	2004	5-HTR2A	rs63311	Yes	
Rybakowski et al.	2006	5-HTR2A	rs63311	Yes	
Brown et al. https://doi.org/10.1016/j.biopsych.2006.04.007	2007	5-HTR1D	rs652783 rs604030 rs674386 rs856510	No	Association analysis
Martásková et al.	2009	5-HTR2A	rs63311	Yes	
Hammer et al https://doi.org/10.1097/FPC.0b013e32833132b3	2009	5-HTR3A	rs1062613 rs1176722	No	Association analysis
Hammer et al. https://doi.org/10.1097/FPC.0b013e32833132b3	2009	5-HTR3B	rs1176744	No	Association analysis
Slof-Op ‘t Landt et al. https://doi.org/10.1111/j.1601-183X.2010.00660.x	2011	5-HTR1D	rs605367 rs6300 rs676643 rs674386	No	Association analysis
Mas et al. https://doi.org/10.1016/j.jpsychires.2012.12.015	2013	5-HTR2A	rs4942577 rs9567733 rs7333412 rs9567736 rs9567737 rs2296972 rs2770298 rs731779 rs1002513 rs927544 rs4942587 rs2296973 rs731245 rs985934	No	Association analysis
Mas et al. https://doi.org/10.1016/j.jpsychires.2012.12.015	2013	5-HTR2C	rs6318 rs518147 rs1801412 rs3813928 rs3813929 rs6311 rs6313	No	Association analysis
Kang et al	2017	5-HTR2A	rs63311	Yes	
Genis Mendoza et al. https://doi.org/10.1002/brb3.1286	2019	5-HTR2A	rs63311	No	Excessively different patients and control samples
Ceccarini et al.	2020	5-HTR2A	rs63311	Yes	

Where possible, gene polymorphisms are identified by the Reference SNP cluster ID (rs). TDT, transmission disequilibrium test

**Table 2:** Biallelic genetic polymorphisms included in the present meta-analysis and numbers of their reports

Gene	Polymorphism	No. of reports
*5-HTR2A*	rs6311 (− 1438G/A)	18
*5-HTR2C*	rs6318 (Cys23Ser)	3

Only polymorphisms investigated in at least three studies are included

The number of reports, 21, exceeds the number of papers, 20, because Karwautz et al. [50] investigated both polymorphisms

**Table 3:** General characteristics of studies included in the present meta-analysis

Author	Year	Nation	Ethnicity	Polymorphism	Genotyping method	Diagnostic criteria	Individual category	Male	Female	Total	Age	BMI	NOS
Hinney et al.	1997	Germany	Caucasian	5-HTR2A rs63311	PCR–RFLP	DSM-IV	Individuals with AN	–	–	100	–	–	7
							Healthy controls	–	–	355	–	–	
Collier et al.	1997	United Kingdom	Caucasian	5-HTR2A rs63311	PCR–RFLP	ICD-10	Individuals with AN	0	81	81	–	–	7
							Healthy controls	138	88	226	–	–	
Campbell et al.	1998	United Kingdom	Caucasian	5-HTR2A rs63311	PCR–RFLP	ICD-10	Individuals with AN		152	152	–	–	6
							Healthy controls		150	150	–	–	
Enoch et al.	1998	U.S.A	Caucasian	5-HTR2A rs63311	PCR–RFLP	DSM-III-R	Individuals with AN	–	–	68	–	–	6
							Healthy controls	–	–	69	–	–	
Sorbi et al.	1998	Italy	Caucasian	5-HTR2A rs63311	PCR–RFLP	DSM-IV	Individuals with AN	0	77	77	24.6 ± 5.8	rAN: 14.9 ± 2.6 pAN: 16.0 ± 1.9	7
							Healthy controls	0	107	107	25.3 ± 5.6	–	
Nacmias et al.	1999	Italy	Caucasian	5-HTR2A rs63311	PCR–RFLP	DSM-IV	Individuals with AN	0	109	109	rAN: 19.2 ± 1.8 pAN: 21.8 ± 4.0	rAN: 13.8 ± 2.5 pAN: 15.6 ± 2.1	6
							Healthy controls	0	107	107	25.3 ± 5.6	–	
Ziegler et al.	1999	Germany	Caucasian	5-HTR2A rs63311	PCR–RFLP	DSM-IV	Individuals with AN	–	–	78	–	–	7
							Healthy controls	–	–	170	–	–	
Ando et al.	2001	Japan	Asian	5-HTR2A rs63311	PCR–RFLP	DSM-IV	Individuals with AN	0	75	75	24.4 ± 5.8	15.7 ± 2.5	6
							Healthy controls	0	127	127	–	–	
Karwautz et al.	2001	United Kingdom	Caucasian	5-HTR2A rs63311	PCR–RFLP	DSM-IV	Individuals with AN	0	44	44	–	–	8
							Healthy controls	0	39	39	–	–	
Nishiguchi et al.	2001	Japan	Asian	5-HTR2A rs63311	PCR–RFLP	DSM-IV	Individuals with AN	–	–	62	25.3 ± 6.2	–	8
							Healthy controls	20	354	374	25.7 ± 10.6	–	
Ricca et al.	2002	Italy	Caucasian	5-HTR2A rs63311	PCR–RFLP	DSM-IV	Individuals with AN	–	–	148	rAN: 19.2 ± 1.8 pAN: 21.8 ± 4.0	rAN: 13.8 ± 2.5 pAN: 15.6 ± 2.1	8
							Healthy controls	–	–	115	26.3 ± 6.1	–	
Kipman et al.	2002	France	Caucasian	5-HTR2A rs63311	PCR–RFLP	DSM-IV	Individuals with AN	–	–	145	–	–	7
							Healthy controls	–	–	98	–	–	
Rybakowski et al.	2003	Poland	Caucasian	5-HTR2A rs63311	PCR–RFLP	DSM-IV	Individuals with AN	–	–	67	–	–	6
							Healthy controls	–	–	114	–	–	
Ricca et al.	2004	Italy	Caucasian	5-HTR2A rs63311	PCR–RFLP	DSM-IV	Individuals with AN	–	–	77	rAN: 23.9 ± 8.4 pAN: 23.9 ± 5.4	rAN: 14.1 ± 2.7 pAN: 15.9 ± 2.4	8
							Healthy controls	–	–	89	21.9 ± 2.5	24.8 ± 2.7	
Rybakowski et al.	2006	Poland	Caucasian	5-HTR2A rs63311	PCR–RFLP	DSM-IV	Individuals with AN	0	131	131	17.6 ± 2.9	–	8
							Healthy controls	0	89	89	20.9 ± 1.6	–	
Martásková et al.	2009	Czechia	Caucasian	5-HTR2A rs63311	PCR–RFLP	ICD-10	Individuals with AN		75	75	25.4 ± 6.2	14.7 ± 1.4	8
							Healthy controls		65	65	25.8 ± 5.1	20.7 ± 1.9	
Kang et al.	2017	China	Asian	5-HTR2A rs63311	SNaP Shot assay	DSM-IV-TR	Individuals with AN	–	–	249	19.1 ± 4.5	–	8
							Healthy controls	–	–	228	20.4 ± 1.5	–	
Ceccarini et al.	2020	Italy	Caucasian	5-HTR2A rs63311	RT-PCR	DSM-5	Individuals with AN	5	306	311	22.4 ± 8.9	14.3 ± 2.0	8
							Healthy controls	38	317	355	27.7 ± 7.9	21.5 ± 2.5	
Karwautz et al.	2001	United Kingdom	Caucasian	5-HTR2C rs6318	PCR–RFLP	DSM-IV	Individuals with AN	0	44	44	–	–	8
							Healthy controls	0	39	39	–	–	
Westberg et al.	2002	Sweden	Caucasian	5-HTR2C rs6318	PCR–RFLP	DSM-III-R	Individuals with AN	0	41	41	–	15.0 ± 2.0	8
							Healthy controls	0	91	91	–	–	
Hu et al.	2003	United Kingdom	Caucasian	5-HTR2C rs6318	PCR–RFLP	DSM-IV	Individuals with AN	–	–	118	–	12.9 ± 2.1	7
							Healthy controls	–	–	316	–	–	

Age:  years ± S.D.; BMI:  kg/m^2^ ± S.D.

NOS: Newcastle–Ottawa Scale; DSM: Diagnostic and Statistical Manual of Mental Disorders; ICD: International Classification of Diseases; PCR–RFLP: polymerase chain reaction-restriction fragment length polymorphism; RT-PCR: real time polymerase chain reaction; SNaP Shot assay: fluorescence labeled single base extension chain termination reaction-based typing assay. M: male; F: female; rAN: patients with restricting type AN; pAN: patients with purging type AN

### Rs6311 polymorphism of *5-HTR2A*

Several studies have evaluated the association of the *5-HTR2A* rs6311 SNP with AN [24–29, 31–39, 57–61]. Ziegler et al. [29] provided genotype frequencies missing in the Hinney et al. [24] study, and correct the data for those miscalculated in the Collier et al. [25] study. We excluded the Gorwood et al. [37] study, included in previous meta-analyses [34, 40], due to virtual calculation of its control participants from non-transmitted alleles in family trios analyzed in TDTs. The Genis-Mendoza et al. study [61] was also excluded due to a considerably smaller number of AN participants (30) compared to control participants (292), a notable difference in age distribution (15 years vs. 30, respectively) and an undefined gender distribution. Details on excluded studies are reported in Table 1.

The eighteen studies included a total of 4926 participants, comprising 2049 patients and 2877 controls), with a NOS quality rating of 7.17 ± 0.75. Genotype and allele frequencies in patients and control cohorts were in HW equilibrium across all studies.

**Fig. 2:**
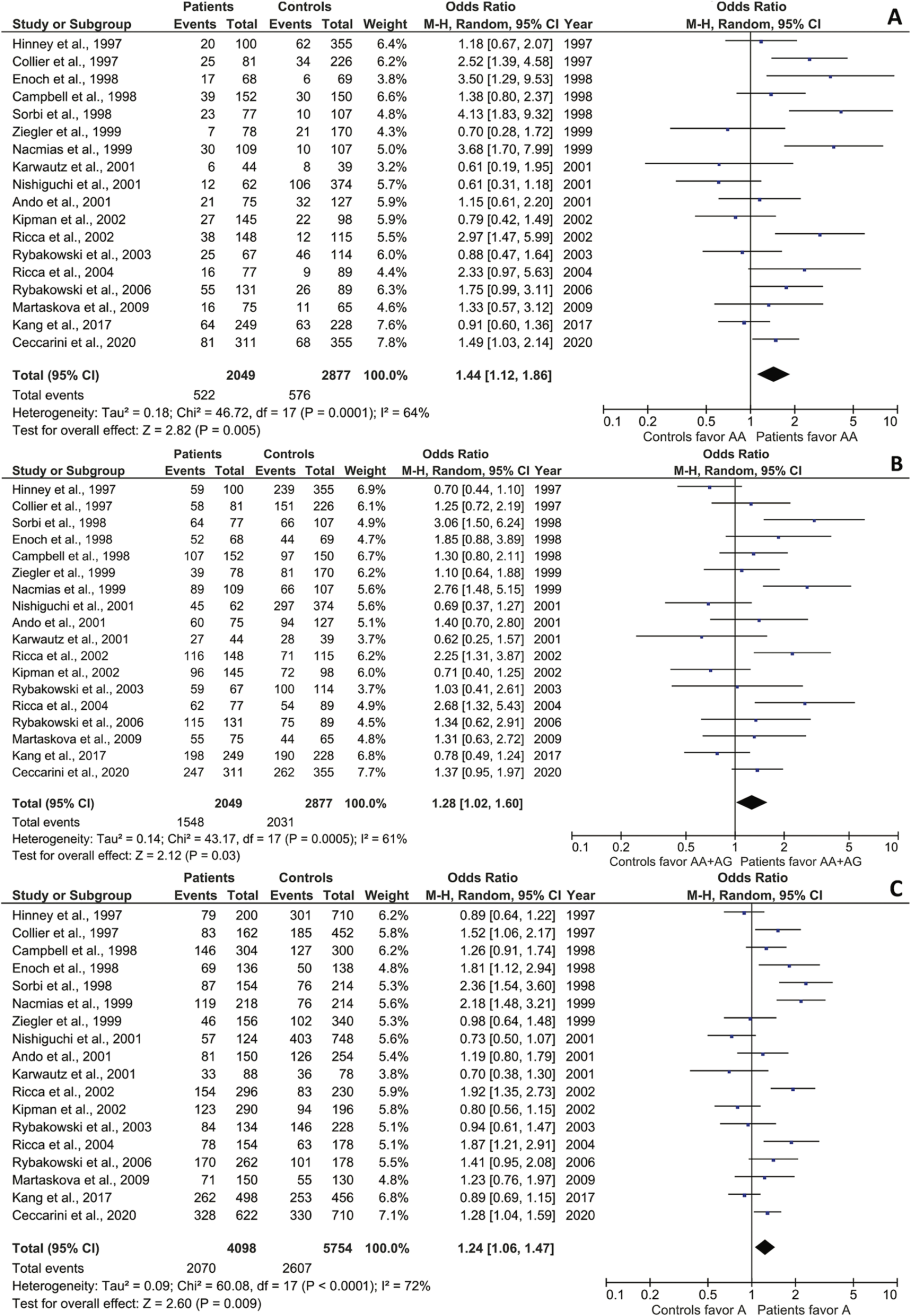
Odds ratios, 95% confidence intervals, and forest plots of individual studies and relative pooled results between AN and the 5-HT2A -1438G/A SNP in different genetic models: **A** recessive (AA vs. AG + GG); **B** dominant (AA + AG vs. GG); and **C**, allele (A vs. G). Egger’s test for publication bias: **A** p = 0.4020; **B** p = 0.4337; **C** p = 0.5992

In an initial global assessment, under the recessive model, we found a small but significant association of the AA genotype with AN (RE, OR 1.44 (95% CI 1.12–1.86); Z = 2.82, p = 0.005) as a risk factor (Fig. 2A), thus as a risk factor. Although substantial effect size heterogeneity and variation were observed (χ^2^ = 46.72; df = 17; p = 0.0001; I^2^ = 64%), the pooled OR did not change significantly in the leave-one-out test, suggesting good result stability. Conversely, according to the dominant model, the pooled AA and AG genotypes did not show an association with AN (RE, OR = 1.28 (95% CI 1.02–1.60); Z = 2.12, p = 0.03) (Fig. 2B), with high heterogeneity and variation among the studies (χ^2^ = 43.17; p = 0.0005; I^2^ = 61%). Under the allele model, the A allele was the predominant one among AN participants, representing a risk factor, with a very small but significant association [RE, OR = 1.24 (95% CI 1.06–1.47); Z = 2.60; p = 0.009]. Again, high heterogeneity and variation were observed (χ^2^ = 60.08; df = 17; p < 0.00001; I^2^ = 72%) (Fig. 2C) although coupled to good result stability, as evidenced by non-significant changes of the pooled OR in the leave-one-out test.

Subsequently, we divided the studies into three geographical regions based on the origins of all cohorts: Central Europe, Britain, Southern Europe, and Asia. NOS quality ratings did not exhibit significant variations among these subgroups (data not shown).

The Central European subgroup comprised 591 patients and 891 controls from Germany [24, 29]; France [33], Poland [35, 59], and Czechia [34]. In this subgroup, no association emerged according to all models: (a) recessive model, [FE, OR = 1.10 (95% CI 0.84–1.43); Z = 0.67; p = 0.50], with low heterogeneity and variation (χ^2^ = 5.31; df = 5; p = 0.38; I^2^ = 6%); (b) dominant model, [FE, OR = 0.92 (0.71–1.17); Z = 0.70, p = 0.48], with low heterogeneity and no variation (χ^2^ = 4.53; df = 5; p = 0.48; I^2^ = 0%); (c) allele model, [FE, OR = 1.00 (95% CI 0.85–1.17); Z = 0.05; p = 0.96, with low heterogeneity and variation (χ^2^ = 5.74; df = 5; p = 0.33; I^2^ = 13%).

The British subgroup [25, 26, 57] comprised 277 patients and 415 controls. No association was observed in this subgroup according to all models: (a) recessive model, [RE, OR = 1.47 (95% CI 0.76–2.85); Z = 1.15; p = 0.25], with low heterogeneity but high variation (χ^2^ = 5.17; df = 2; p = 0.08; I^2^ = 61%); (b) dominant model, [FE, OR = 1.16 (95% CI 0.83–1.62); Z = 0.86, p = 0.39], with very low heterogeneity and variation (χ^2^ = 2.01; df = 2; p = 0.37; I^2^ = 1%); (c) allele model, [RE, OR = 1.19 (95% CI 0.83–1.70); Z = 0.96; p = 0.34], with low heterogeneity but substantial variation (χ^2^ = 4.46; df = 2; p = 0.11; I^2^ = 55%).

The Southern European subgroup comprised Italian studies [28, 31, 39, 58, 60] totaling 722 patients and 733 controls. Significant associations were observed in this subgroup according to all models: (a) recessive model, with a medium association [RE, OR = 2.55 (95% CI 1.62–4.02); Z = 4.05, p < 0.0001], low heterogeneity but substantial variation (χ^2^ = 9.08; df = 4; p = 0.06; I^2^ = 56%); (b) dominant model, with a small association [FE, OR = 1.98 (95% CI 1.56–2.51); Z = 5.66, p < 0.00001], low heterogeneity and some variation (χ^2^ = 7.41; df = 4; p = 0.12; I^2^ = 46%); and (c) allele model, with a small association [RE, OR = 1.82 (95% CI 1.41–2.37); Z = 4.55; p < 0.00001], some heterogeneity but substantial variation (χ^2^ = 10.93; df = 4; p = 0.03; I^2^ = 63%). Upon conducting leave-one-out tests, exclusion of the Ceccarini et al. [39] study, conducted in Umbria, resulted in an increase in association values and a decrease in heterogeneity and variation among the four other studies, all conducted in Tuscany, comprising 411 patients and 378 controls, according to all models: (a) recessive model [FE, OR = 3.23 (95% CI 2.18–4.78); Z = 5.86; p < 0.00001, χ^2^ = 1.04; df = 3; p = 0.79; I^2^ = 0%]; (b) dominant model [FE, OR = 2.62 (95% CI 1.91–3.59); Z = 5.97; p < 0.00001, χ^2^ = 0.52; df = 3; p = 0.91; I^2^ = 0%]; (c) allele model [FE, OR = 2.07 (95% CI 1.69–2.52); Z = 7.18; p < 0.00001, χ^2^ = 0.80; df = 3; p = 0.85; I^2^ = 0%]. Exclusion of other studies did not result in substantial variations.

The Asian subgroup comprised Japanese [32, 36] and Chinese Han [38] cohorts, totaling 386 patients and 729 controls. No association emerged in this subgroup according to all models: (a) recessive model [FE, OR = 0.87 (95% CI 0.64–1.18); Z = 0.88; p = 0.38], with very low heterogeneity and no variation (χ^2^ = 1.89; df = 2; p = 0.39; I^2^ = 0%); (b) dominant model, [FE, OR = 0.86 (95% CI 0.62–1.19); Z = 0.89, p = 0.37], with small heterogeneity and some variation (χ^2^ = 2.64; df = 2; p = 0.27; I^2^ = 24%); (c) allele model, [FE, OR = 0.90 (95% CI 0.75–1.09); Z = 1.06; p = 0.29], with low heterogeneity and some variation (χ^2^ = 3.05; df = 2; p = 0.22; I^2^ = 34%).

The Enoch et al. [27] study, performed on North American (U.S.A.) participants, suggested a positive association but was not included in any subgroup.

For all subgroups and across all models, except for the Italian subgroup, the leave-one-out tests did not alter the pooled OR significantly, indicating the stability of the results.

In summary, the geographic subgroup analysis revealed an association of the *5-HTR2A* rs6311 polymorphism according to all models only in Italian studies, particularly within the Tuscany cohorts. This suggests a potential regional specificity for the association of this polymorphism and AN.

## Rs6318 polymorphism of *5-HTR2C*

**Fig. 3:**
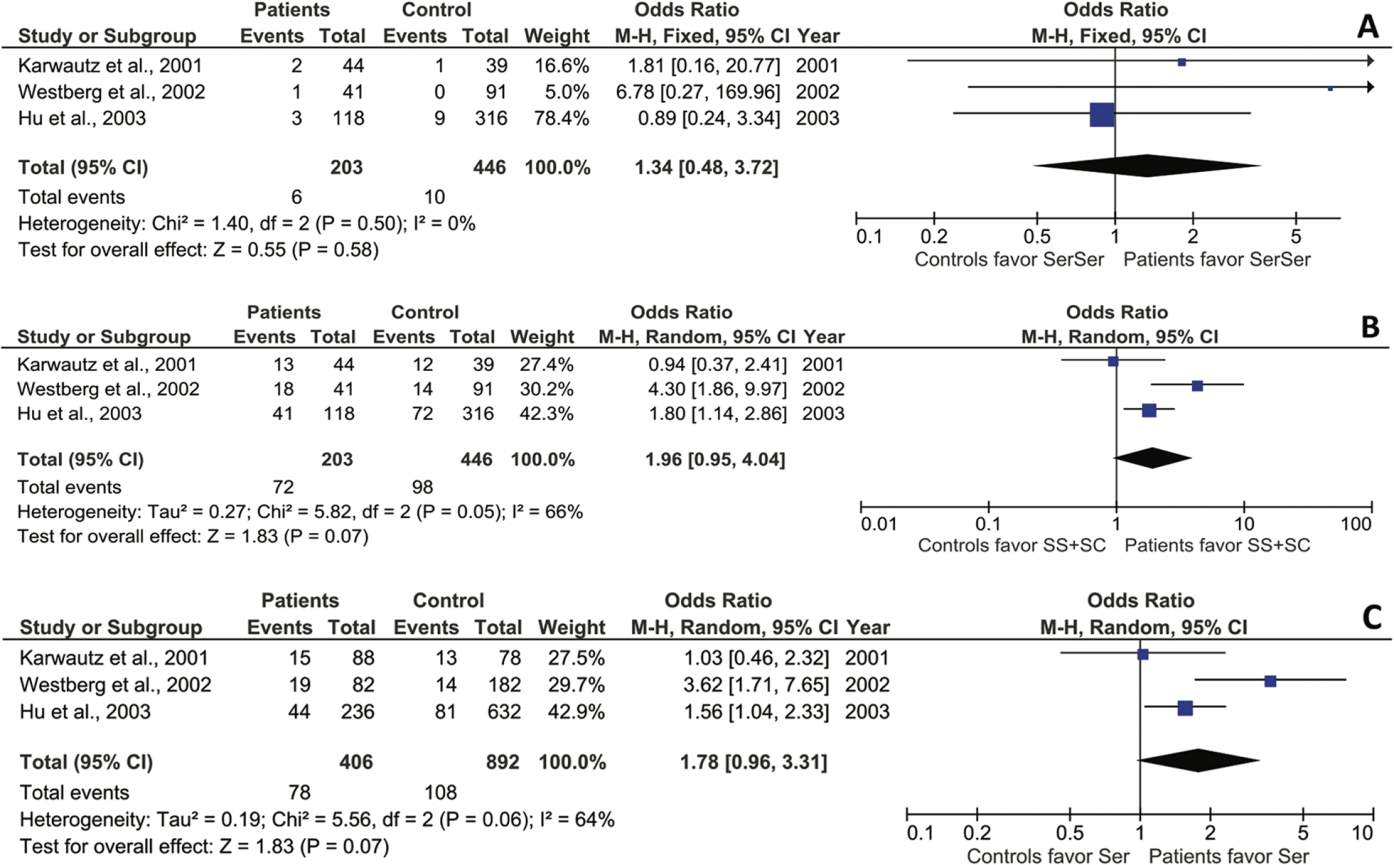
Odds ratios, 95% confidence intervals, and forest plots of individual studies and relative pooled results between AN and the 5-HT2C Cys23Ser SNP in different genetic models: **A** recessive [SerSer (SS) vs. SerCys (SC) + CysCys (CC)]; B, dominant (SS + SC vs. CC); and **C** allele (S vs. C). Egger’s test for publication bias: **A** p = 0.1650; **B** p = 0.9612; **C** p = 0.8586

The rs6318 (Cys23Ser) SNP of 5*-HTR2C* has been investigated in three studies [57, 62, 63], with 649 total participants, comprising 203 patients and 446 controls. The NOS quality rating of the studies was 7.67 ± 0.58, and genotype and allele frequencies were in HW equilibrium across all cohorts. Under the recessive model, the SerSer genotype was not associated with AN [FE, OR 1.34 (95% CI 0.48–3.72); Z = 0.55, p = 0.58] (Fig. 3A), with negligible heterogeneity and variation (χ^2^ = 1.40; df = 2; p = 0.50; I^2^ = 0%). Under the dominant model, presence of the Ser allele displayed a slight tendency towards association with AN as a risk factor [RE, OR = 1.96 (95% CI 0.95 – 4.04); Z = 1.83, p = 0.07] (Fig. 3B), albeit with moderate heterogeneity but high variation (χ^2^ = 5.82; df = 2; p < 0.05; I^2^ = 66%). Similar results were observed under the allele model [RE, OR = 1.78 (95% CI 0.96 – 3.31; Z = 1.83; p = 0.07], suggesting a tendency for the association of the Ser allele with AN as a risk factor, but with moderate heterogeneity and high variation (χ^2^ = 5.56; df = 2; p = 0.06; I^2^ = 64%) (Fig. 3C). The available evidence does not support the existence of an association between this SNP and AN.

## Discussion

In this paper, we describe meta-analyses of candidate gene studies on AN, focusing on *5-HTR2A* rs6311 and *5-HTR2C* rs6318 SNPs, allowing a reevaluation of previously reported information for the former and providing novel results for the latter.

### Rs6311 polymorphism of *5-HTR2A*

*5-HTR2A,* mapped on chromosome 13q14-q21, has been implicated in various neuropsychiatric disorders, including depression [64], and central nervous system pathologies, such as epilepsy [65]. The rs6311 (-1438G/A) SNP is located in the distal promoter region of *5-HTR2A.* Its functional role remains uncertain: while some studies have linked the A allele with increased transcriptional [66, 67] and translation efficiency [68], as well as higher serotonin binding [69, 70], compared to the G allele, these findings have not been replicated in other studies [71, 72].

Results of numerous reports and previous meta-analyses [29, 30, 34, 40] have suggested a potential association of this SNP with AN, depending on the geographic distribution of the cohorts involved.

Our meta-analysis incorporates three additional studies to those of Yan et al. [40], and specifically addresses this issue. Upon global analysis, results of the existing studies confirm the presence of an association according to the recessive and allele models, but not the dominant model, with the AA genotype and A allele representing risk factors for AN. In this assessment, taking into account participant numbers and ORs, the estimated attributable risk in the development of AN is 7.8% for the AA genotype and 9.7% for the A allele [73].

However, our geographical partition reveals the presence of a clear association only in the Italian subgroup. In its cohorts, the estimated attributable risk for the AA genotype and A allele in the development of AN is higher, at 15.7% and 10.1%, respectively [73]. Furthermore, a more robust association is observed in the Tuscany cohorts, suggesting a dependence of the association on specific geographic locations and differences in ethnic groups.

In summary, our findings indicate that the positive association observed globally in present and previous meta-analyses originates from a limited number of studies, mainly conducted in Italy. This observation emphasizes the need for further investigations, including molecular and cellular studies focused on gene expression and receptor-mediated transduction signaling, respectively, to assess the potential functional role of the rs6311 SNP of *5-HTR2A* in the integration of neuronal activity.

### Rs6318 polymorphism of *5-HTR2C*

The *5-HTR2C* gene, mapped on chromosome Xq24, has attracted attention due to pharmacological evidence suggesting its involvement in food intake regulation and AN in both rodents [74, 75] and humans [76, 77].

The rs6318 (Cys23Ser) SNP of 5*-HTR2C* involves a cysteine (Cys) to serine (Ser) substitution at position 23 in the N-terminal extracellular domain of the receptor [78]. Studies exploring the association of this SNP with AN emerged in 2001, supported by the finding of serotonin-binding differences between the two allele variants [79], but waned after 2003, likely due to observations of no functional differences between the alleles [80]. Although our meta-analysis does not reveal an association with AN, recent research has identified pharmacological and subcellular localization differences between them [81]. Consequently, further investigations on this SNP promise for uncovering significant insights.

## Conclusions

This meta-analysis elucidates potential roles of *5-HTR2A* rs6311 and *5-HTR2C* rs6318 SNP*s* in the etiology of AN. While evidence points to an association between the *5-HTR2A* rs6311 polymorphism and AN in Italian cohorts, no associations are observed from other geographic regions. These findings unify the results of all previous meta-analyses and support the conclusions of recent reviews [82, 83]. They underscore the notion that this polymorphism should be viewed within a multifactorial inheritance framework that accounts for the interplay of multiple genes and potential environmental factors in the development of a psychiatric disorder.

As for the *5-HTR2C* rs6318 polymorphism, no evidence for an association with AN was observed.

In conclusion, while the involvement of serotonin in regulating eating behaviors and contributing to the development of AN is acknowledged, attributing this role to individual polymorphisms of serotonin receptor genes remains ambiguous. In the future, multiple genetic factors involved in AN will likely be identified through genome-wide investigations. Additionally, analysis of the interaction of multiple genes, such as those encoding for 5-HTT and the norepinephrine transporter [84] or 5-HTT and monoamine-oxidase [85], would offer further insights. Lastly, it will be imperative to acquire more information on the etiologic roles of psychological, environmental, familial and social factors [86]. In this context, the advancement of epigenetic research holds significance, by allowing the evaluation of gene x environment interactions potentially involved in AN.

In a bio-psycho-social framework, identifying genuine genetic and epigenetic markers would be valuable in an integrated strategy aimed at early risk detection. Under a pharmacogenomic approach, this could also facilitate the development of improved standard or nutritional therapies tailored to individual responses. From a clinical perspective, understanding the genetic factors associated with AN could offer support to patients and their families, potentially alleviating the burden of blame often linked with the disorder [87]. However, no definitive clinical implications can be drawn from individual serotonin gene polymorphisms considered in the present meta-analysis. Consequently, engaging in discussions about the genetics of AN with affected individuals and their families remains challenging, and the possibility of implementing prevention strategies or genomic approaches to the pathology appears to be postponed into the future.

## Strength and limits

This meta-analysis evaluates the outcomes of serotonin candidate gene studies on AN, focusing on the *5-HTR2A* rs6311 polymorphism and the *5-HTR2C* rs6318 polymorphism. It has various strengths: a) it was conducted by an up-to-date search of articles published between 1997 and 2022; b) article selection was rigorous, employing specific inclusion/exclusion criteria; c) all results underwent statistical correction for multiple testing; and d) it includes a comprehensive geographic evaluation of effect sizes for the *5-HTR2A* rs6311 polymorphism.

Similar to all meta-analyses, we acknowledge the possibility of missing publications, despite our rigorous search strategy. Other limitations are inherent of published reports, including small sample sizes of many studies, relatively low numbers of total individuals, subgroup analyses limited to geographic regions.

Finally, this meta-analysis does not differentiate between the binge eating/purging subtype and restricting subtype of AN, since this issue is reported only in some studies. Increasing the number of genetic studies focusing on these two diagnoses would be advisable to gain deeper insights into these phenotype variants.

## What is already known on this subject?

Existing research of the subject has seen several case–control candidate gene studies examining the association between serotonin receptor genes and AN from 1997 to 2022. However, these studies have yielded inconsistent results. Additionally, various meta-analyses focusing on the *5-HTR2A* rs6311 polymorphism have been conducted between 1999 and 2021. Despite these efforts, methodological and/or statistical weaknesses in these studies have led to conflicting conclusions, raising doubts about the presence of a definitive association between this polymorphism and AN. Conducting an updated and rigorous meta-analysis to gather further information on the *5-HTR2A* rs6311 polymorphism, and potentially other serotonin receptor gene polymorphisms, could provide valuable insights into their involvement in AN.

## What this study adds?

This meta-analysis focuses on the association of the *5-HTR2A* rs6311 polymorphism and the *5-HTR2C* rs6318 polymorphism with AN. The findings indicate the absence of association between the *5-HTR2A* rs6311 polymorphism and AN when analyzed globally or in various geographic subgroups but pinpoint presence of association in an Italian subgroup, primarily represented by cohorts from Tuscany. They also report no evidence of an association between the *5-HTR2C* rs6318 polymorphism and AN.

With the exclusion of the results relative to the *5-HTR2A* rs6311 polymorphism in the Italian region that would need further examination, these observations underscore the notion that an etiological role in AN cannot be attributed to individual polymorphisms of serotonin receptor genes. This strengthens the importance of exploring multifactorial inheritance paradigms to understand factors involved in the disorder.

Under a clinical perspective, these findings do not allow to conceive, at present, the implementation of prevention strategies or genomic approaches in supporting and treating subjects with AN.

## Data Availability

Data used for the present meta-analysis are available from the corresponding author upon reasonable request.
